# Midwifery

**Published:** 1838-07

**Authors:** 


### MIDWIFERY.

*Report of two Cases of Pregnancy requiring Incision of the Os Uteri to allow Delivery.*

I. By Dr. Burdach, of Finsterwalde. *Cartilaginous State of the Os Uteri.*

A strong robust woman, æt. 28, pregnant for the first time, felt the pains commence on the 24th of June, and the membranes broke in the course of the day. The midwife was unable to reach the os uteri, and, although the pains in some degree continued, no progress had been made towards delivery on the 26th. Borax, in doses of six grains, were then administered, with a view of increasing the pains; but, although this object was attained, no further progress was made. On the 27th, Dr. Burdach was called in; and found, on examination, the anterior lip of the os uteri hard and callous. Vapour-baths, and the application of an opiate ointment to the os uteri, were ordered, and continued till the forenoon of the next day; when matters remaining precisely in the same condition, an incision of one inch and a half was made into the anterior lip. Only a small quantity of blood escaped. Doses of eight grains of borax, with one of musk, were given to increase the pains: but, matters proceeding slowly, the forceps were applied, and a dead child extracted, without any tearing of the incision of the os uteri. The wound healed kindly, and the woman made a good recovery.

II. By Dr. Gruhn, of Reppen. *Prolapsus Uteri.*

A strong, robust woman, æt. 28, had enjoyed good health during the first four months of pregnancy. At this time, whilst raising a heavy weight, she had the feeling as if something had given way in the abdomen, and shortly afterwards the uterus began to protrude from the vagina, and continued to do so till the end of her pregnancy. When Dr. Gruhn arrived, she had already been in labour for thirty-six hours, and twenty-four hours had elapsed since the waters had come away. The woman was lying on her back in bed, in a very low, dispirited state, with the uterus, containing the fœtus, between her thighs: its longitudinal diameter was equal to about six inches, and the transverse diameter to eight inches. The vertex of the child presented, and the os uteri had dilated to the size of half a dollar. All attempts to dilate it further proving abortive, an incision, three inches in length, was made into one side of the os uteri, and a fully developed though dead child extracted. The placenta followed immediately, accompanied by a gush of blood, which greatly reduced the patient. The flooding was stopped by injections of cold water into the uterus; and the accoucheur, introducing his hand into its cavity, proceeded to reduce it. The operation was successful: no further flooding followed, the milk was secreted, and the lochial discharge appeared. The woman was recommended to wear a pessary, but neglected to do so on the plea of inconvenience. *Medicinische Zeitung.* 1837. No. 39.

---

### *Singular Case of Abortion.*

Dr. Erbkam, second physician of the Obstetric Clinical Institute of Berlin, has described a very remarkable case of abortion in the fourth month, where the motions of the fœtus continued strong for some time after birth. Finding, upon being expelled, that it moved, he tied the cord, and placed it in warm water. The movements, which consisted in drawing up the feet and arms, turning the head from one side to another, and opening the mouth as if endeavouring to breathe, continued for half an hour. The action of the heart was visible for ten minutes after all other movements had ceased: as soon as fresh warm water was added, these motions again returned. The length of the fœtus was six inches, its weight eight ounces; the head was sufficiently developed, and the cranial bones considerably advanced in the process of ossification; the eyes were closed. From a superficial inspection of the external genitals, it might have been taken for a female fœtus, there being well-marked labia, between which a large clitoris projected: on opening the abdomen, however, the testes were found in its cavity. The fœtus was shewn to Professor J. Müller, who expressed it as his conviction that the fœtus was not more than four months advanced.

*Neue Zeitschrift für Geburtskunde.* B. v. H. 2.

---

### *Inversion of the Uterus resembling Polypus.* By M. Fleury, Jun.

A woman, aged forty-six, mother of five children, and who had always had easy deliveries, enjoyed good health up to the occurrence of the disease under which she now labours. Three years since a tumour first made its appearance in the vagina; this was preceded by a discharge of a red colour which had existed eighteen months. When the tumour first made its appearance externally, a leucorrhœal discharge took place so abundant as speedily to exhaust the strength of the patient. A portion of the tumour was excised in the month of August, 1836, (about two years after the first appearance of the disease,) and she experienced great relief; the discharge diminished greatly, but the amendment was of short duration; and at the same time that the tumour regained its former size the discharge re-appeared in the same quantity as before. In the month of February, 1837, she was received into the Hôtel Dieu at Clermont. At that time her constitution seemed exhausted, her face bore traces of great suffering, and her skin had that strongly marked straw colour, which, at first sight, seemed to be the effect of cancerous cachexy. The finger, introduced into the vagina, discovered at once a pyriform tumour with its base inferior. The circumference both of the base and pedicle was easily traced, but when the finger reached the summit of the tumour it was found quite impossible to penetrate the uterus. It seemed as if the pedicle was inserted into the whole circumference of the os tincæ, or that it had formed attachments to it. Its texture was firm and resisting, and it was quite insensible to the touch. The red discharge still continuing, and further delay appearing dangerous to the patient, an operation was determined upon. The patient was placed upon a low table, in the same position as in the operation for the stone. A pair of uvula forceps was then introduced into the vagina, and passed upon the fore-finger of the left hand up to the tumour, which was easily seized and brought to the os externum by gentle and continued pulling. Its colour was a pale red, its surfaces quite smooth, and its form that of the body of the uterus. The thumb and fore-finger carried beyond the pedicle of the tumour were separated from each other only by the sides of the vagina applied the one against the other. No doubt remained that this tumour was the inverted uterus, for had it been a polypus, the neck of the uterus would have been found around and above the pedicle. On applying the finger to the upper part of the tumour the pulsations of arteries, apparently of considerable size, were plainly perceived. The exposed surface, then, was the internal face of the uterus, which had become external and was the source of the sanguineous discharge. The patient made no complaint and suffered no pain during the operation; even when the uterus was between the blades of the forceps its structure seemed quite insensible. The diagnosis having been thus clearly established, what treatment was to be pursued? Ought an attempt to be made to replace the inverted viscus? was a ligature to be applied? or ought the uterus to be left to itself? The last alternative was adopted; a little charpie was introduced into the vagina with a view of exercising a slight pressure on the uterus, and thus lessening the size of the tumour. The result of this simple treatment, however, could not be known, as the patient soon left the hospital. The question naturally suggests itself, what was the nature of the tumour which was extracted in the summer of 1836? Was it a polypus attached to the body of the uterus or a portion of the uterus itself? Had it been a portion of the uterus itself that organ must have lost its regularity, and had it been a polypus some traces of cicatrization might be expected: they did not, however, exist. It is most probable that the tumour removed was a polypus, and that the attempts to extract it had caused inversion of the uterus.

[This case furnishes a marked exception to a diagnostic rule generally laid down, that in inversion of the uterus the tumour is painful to the touch, in case of polypus insensible. The sensibility of the uterus is perhaps overrated: a case related by Dr. Sparman in his travels in the Gape of Good Hope would lead us to think that it has a very low degree of sensibility. He says that he met with a native who had had prolapsus of the uterus, and finding the tumour troublesome had pulled it away piece-meal. We will not pretend to fix the degree of credit which should attach to narratives of cases of disease contained in books of travels, but as Sparman was a physician we feel justified in quoting his authority.]

*La Presse Médicale.* No. 58. Tom. i. 1837.

---

### *Remarkable Case of Embryotomy on account of Exostosis of the Pelvis.* By Dr. KYLL, of Cologne.

THE patient was a robust peasant woman, æt. forty-five, mother of seven children: her former labours had presented nothing unusual, except that in her last, which occurred in her forty-second year, medical assistance had been required to remove the after-birth, which the practitioner declared had formed an usually firm adhesion to the uterus. She experienced no ill effects from its extraction, beyond suffering a good deal of pain at the time. On the sixth day after labour, she was seized with feverish symptoms and violent pain, at the spot where the placenta had been removed. The attack yielded to proper treatment, but she continued feverish at night, with perspirations, bowels frequently deranged, difficulty in passing water, with severe pain in the abdomen, especially when she tried to stand on the right leg. An abscess formed in the right groin, which was opened, and discharged a large quantity of pus. Her recovery was very slow; she became extremely emaciated, and did not leave her bed for seven months. On getting up for the first time, it was observed that she limped, and the right leg was evidently shorter than the left; but, with this exception, she completely recovered her health and strength, and again became pregnant three years after. On labour coming on, the midwife could reach no presenting part; but, after some hours, when the os uteri had dilated considerably, a foot presented, and shortly after the other descended also; and, after long and severe pains, they approached the os externum, but no extractive force could make them advance further. During the night, a coil of the cord prolapsed: it was without pulsation; but it was not until the following morning that the patient could be induced to permit a medical man to be sent for.

On his arrival, Dr. Kyll found, upon external examination, the uterus very high and inclined to the left side. On examination per vaginam, he found the child resting with the hips on the brim of the mother's pelvis, and completely wedged fast by a hard tumour, which sprung from the upper part of the sacro-iliac symphisis: it was about the size of a small hen's egg and immoveable. Below this projection, the pelvis was sufficiently spacious. From these circumstances, it seemed evident that, during her last confinement, she had suffered from pelvic abscess, which had, in all probability, caused this exostosis. The child being evidently dead, the perforation was determined on.

Dr. K. first of all endeavoured to bring the pelvis of the child through the superior aperture, by pulling firmly at the feet, and succeeded at length in bringing it as far as the breast, beyond which it would not stir: he therefore opened the abdominal cavity with a bistoury, passed his hand through the diaphragm, and evacuated the contents of the thorax. The thorax now descended, but the two arms had become turned up on each side of the head. As there was not sufficient space to bring down the arms, Dr. Kyll removed them at the shoulder-joints with a pair of scissors: he then perforated the head at the occiput, in the hopes of bringing it down when the bones collapsed. As, however, he found that this was impossible, owing to the breadth of the basis cranii, the only means of bringing it away would be by reducing the size of the head as much as possible, and then bringing it down with the occiput foremost, and the face upwards, through the left oblique diameter of the brim.

The head was very far from being sufficiently diminished by perforation; the narrow space required that its perpendicular diameter should be lessened, in order that the vertex should not be stopped by the anterior wall of the pelvis. In order to effect this, it became necessary to remove the body of the child, in order to gain sufficient space to get at the head. This was done close to the foramen magnum, by means of the scissors, leaving the head, which rolled about upon the brim of the pelvis with every movement of the mother. To diminish the size of the head, Dr. K. introduced his right hand to the left side of the pelvis, fixed the head by the left hand on the outside, and placed it with the face looking upwards, the sagittal suture turned to the left, and then pushed it as much as possible to the right side of the pelvis to gain sufficient space for his right hand; and, in order to fix the head in this position, he passed a sharp hook into the foramen magnum, and held it with the left hand. He now introduced a bistoury with his right hand, passed it up to the anterior fontanelle, which he cut through, and then divided the cranial integuments the whole length of the sagittal suture. Having withdrawn the knife, he removed first one and then the other parietal bone: this was the most difficult part of the whole operation, but it succeeded completely. He took this opportunity of removing the rest of the brain; and, having thus lessened the head, he placed it in the left oblique diameter of the pelvis, with the face upwards, and brought it through the pelvis without any exertion. The uterus contracted well, the placenta was easily expelled, and the patient recovered very quickly.

Immediately after the labour, he examined the tumour carefully, and felt convinced that it was an exostosis growing from the right sacro-iliac symphisis.

*Neue Zeitschrift für Geburtskunde.* B. v. H. 1.

